# FCC to BCC transformation-induced plasticity based on thermodynamic phase stability in novel V_10_Cr_10_Fe_45_Co_x_Ni_35−x_ medium-entropy alloys

**DOI:** 10.1038/s41598-019-39570-y

**Published:** 2019-02-27

**Authors:** Y. H. Jo, W. M. Choi, D. G. Kim, A. Zargaran, S. S. Sohn, H. S. Kim, B. J. Lee, N. J. Kim, S. Lee

**Affiliations:** 1 Center for High Entropy Alloys, Pohang University of Science and Technology, Pohang, 790-784 Korea; 2 Graduate Institute of Ferrous Technology, Pohang University of Science and Technology, Pohang, 790-784 Korea

## Abstract

We introduce a novel transformation-induced plasticity mechanism, *i*.*e*., a martensitic transformation from fcc phase to bcc phase, in medium-entropy alloys (MEAs). A VCrFeCoNi MEA system is designed by thermodynamic calculations in consideration of phase stability between bcc and fcc phases. The resultantly formed bcc martensite favorably contributes to the transformation-induced plasticity, thereby leading to a significant enhancement in both strength and ductility as well as strain hardening. We reveal the microstructural evolutions according to the Co-Ni balance and their contributions to a mechanical response. The Co-Ni balance plays a leading role in phase stability and consequently tunes the cryogenic-temperature strength-ductility balance. The main difference from recently-reported metastable high-entropy dual-phase alloys is the formation of bcc martensite as a daughter phase, which shows significant effects on strain hardening. The hcp phase in the present MEA mostly acts as a nucleation site for the bcc martensite. Our findings demonstrate that the fcc to bcc transformation can be an attractive route to a new MEA design strategy for improving cryogenic strength-ductility.

## Introduction

Development, transportation, and preservation of resources through recent scientific and technological advances are increasingly demanding novel metals to be applied in extreme and risky environments. Unceasing efforts have been made to improve both strength and ductility^1–4^, but cryogenic environments often cause a severe deterioration in ductility because of low damage-tolerance capacities and crystallographic problems^5,6^. In this respect, recently developed face-centered-cubic (fcc) high-entropy alloys (HEAs) present excellent tensile properties and exceptional fracture toughness by forming mechanical nano-twins at cryogenic temperature^7,8^. For the further improvement of mechanical properties, studies on various strengthening mechanisms such as (1) high lattice distortion and solid-solution strengthening by utilizing multi-components^9,10^, (2) grain refinement^11,12^, and (3) precipitation strengthening by adding Al, Mo, and interstitial C^13–15^ have been actively conducted.

The deformation-induced martensitic transformation, which has been widely employed in numerous high-strength structural alloys, is also introduced into HEAs. For example, (1) fcc to hexagonal-close-packed (hcp) transformation in FeMnCoCr, CoCrFeMnNi and CrCoNi alloys^1,16–18^, (2) body-centered-cubic (bcc) to orthorhombic transformation in Al_0.6_CoCrFeNi alloy^19^, and (3) bcc to hcp transformation in TaHfZrTi alloy^20^ were reported. It is known from advanced high-strength steel researches that the fcc to bcc transformation can be more suitable for improving the strength and toughness balance than the fcc to hcp transformation^21,22^. However, there are no studies on the fcc to bcc transformation in HEA research areas.

In this study, thus, we exploit the fcc to bcc phase transformation in MEAs for the first time. We newly design a VCrFeCoNi MEA in consideration of thermal and mechanical stabilities of phases to generate the fcc to bcc transformation by utilizing thermodynamic calculations. We reduce a difference in Gibbs free energies between bcc and fcc phases by careful adjustment of constituent elements of the MEA in order to obtain a stable bcc phase. The resultantly formed bcc martensite favorably contributes to the TRansformation-Induced Plasticity (TRIP), thereby leading to a significant enhancement in both strength and ductility as well as strain hardening.

## Results

### Alloy design based on CALPHAD approach

To generate the fcc to bcc martensitic transformation, we adopt the following strategies: (1) to obtain a single fcc phase in the as-annealed state and (2) to increase the thermal stability of bcc phase at low temperature. It is well known that a key parameter for designing metastable MEAs as well as conventional structural alloys which show the TRIP behavior, *i*.*e*., the fcc to bcc transformation, is the stacking fault energy (SFE) of fcc. Though the SFE contains a term expressed by a difference in Gibbs free energies between hcp and fcc phases^6,23,24^, the fcc to bcc transformation is also analyzed by the SFE. In this study, however, we reveal that a difference in Gibbs free energies between bcc and fcc phases can be a critical parameter for the fcc to bcc martensitic transformation. The phase equilibrium and stability were calculated by using a Thermo-Calc^25^ software along with the thermodynamic database TCFE2000 and its upgraded version^26–28^. This CALPHAD approach is much more effective than trial-and-error-based conventional alloy-design approaches consuming a lot of costs and time^29,30^. Thermodynamic calculations based on the present database are not accurate below 0 °C, but the 0 °C-calculations can be reasonably acceptable for the stability evaluation at cryogenic temperature^6^.

Alloying elements such as Fe, Cr, and V are considered firstly in the present MEA design. Fe is a cost-effective element in fcc MEA systems, and its increase helps to generate the formation of bcc or hcp phase. We set the Fe content to be 45 at.%, based on alloy-design conditions underlying that complex concentrated alloys (CCA) such as HEA and MEA are not Fe-based alloys but multi-principal-element alloys^31–33^. Cr which improves the corrosion resistance is set to be 10 at.%. The third candidate element is 10 at.% of V. V is usually utilized in bcc refractory HEA fields^34^, but can also be added to single-fcc-phase HEAs if the formation of sigma phase is suppressed^35^. V possesses a larger negative mixing enthalpy and a resultantly stronger bonding with Fe, Co, and Ni than bonding between Cr, Fe, Co, and Ni^36^. A substantial solid-solution hardening effect can also be expected by a high lattice distortion^37^ because the atomic size of V is considerably larger than that of Cr, Fe, Co, and Ni. In order to obtain a single fcc phase, thus, careful element control through a thermodynamic approach is primarily required.

**Figure 1:**
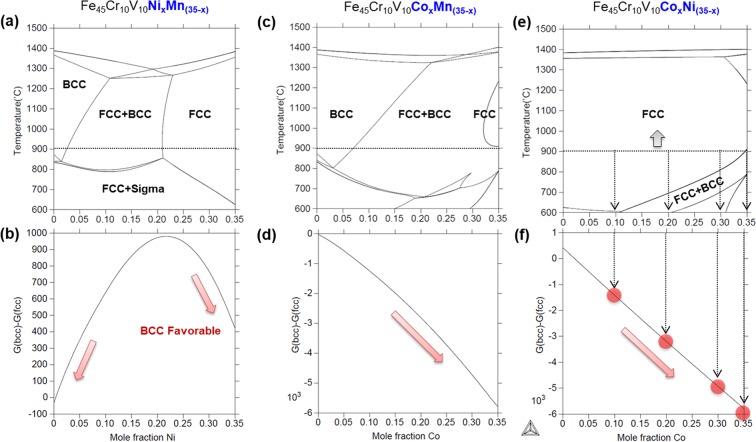
Thermodynamic design approach of Fe_45_Cr_10_V_10_Ni_x_Mn_(35−x)_ alloy. (**a-f**) Equilibrium phase diagrams and ΔG_bcc-fcc_ data of Fe_45_Cr_10_V_10_Ni_x_Mn_(35−x)_, Fe_45_Cr_10_V_10_Co_x_Mn_(35−x)_, and Fe_45_Cr_10_V_10_Co_x_Ni_(35−x)_ systems in the temperature range of 600 °C~1500 °C. In order to obtain a stable single fcc phase, to control the stability, and to confirm our design strategy, four alloying compositions are chosen at 10, 20, 30, and 35 at.% of Co content in the Fe_45_Cr_10_V_10_Co_x_Ni_(35−x)_ system.

The rest candidate elements to obtain a single fcc phase are Ni, Mn, and Co. Figure 1(a) shows an equilibrium phase diagram of Fe_45_Cr_10_V_10_Ni_x_Mn_(35−x)_ in the temperature range of 600–1500 °C. In this system 20 at.% or higher of Ni is required at least to obtain a single fcc phase at 900 °C. Figure 1(b) shows stability of bcc and fcc, *i*.*e*., a difference in Gibbs free energies between bcc and fcc (ΔG_bcc-fcc_) at 0 °C. The lower ΔG_bcc-fcc_ indicates, the more stable bcc phase. This diagram reveals that the sole addition of Ni or Mn favors the formation of bcc phase. The decrease in ΔG_bcc-fcc_ is not quite large (~600 J·mol^−1^) at 20 at.% or higher of Ni, indicating the Fe_45_Cr_10_V_10_Ni_x_Mn_(35−x)_ system is not desirable for the present aim.

According to the same calculation for the Fe_45_Cr_10_V_10_Co_x_Mn_(35−x)_ system (Fig. 1(c,d)), the increase in Co content dramatically reduces the ΔG_bcc-fcc_. However, a single fcc region is limited, and the bcc or other intermetallic phases can be formed during thermo-mechanical processes. For the Fe_45_Cr_10_V_10_Co_x_Ni_(35−x)_ system (Fig. 1(e,f)), the fcc region considerably expands and the stability of bcc phase can increase with Co addition. In order to obtain a stable single fcc phase, to control the stability, and to confirm our design strategy, thus four alloying compositions are chosen at 10, 20, 30, and 35 at.% of Co content in the Fe_45_Cr_10_V_10_Co_x_Ni_(35−x)_ system. The MEAs whose Co contents are 10, 20, 30, and 35 at.% are referred to as ‘10Co’, ‘20Co’, ‘30Co’, and ‘35Co’, respectively, for convenience.

### Microstructure

**Figure 2:**
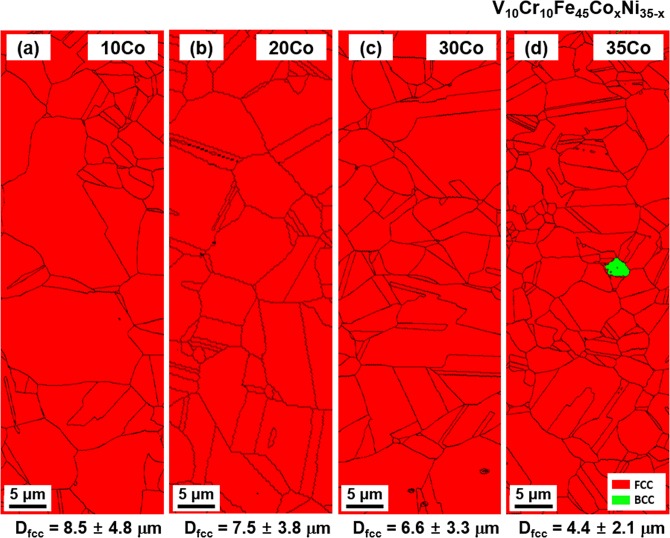
As-annealed microstructure. EBSD phase maps of the as-annealed (**a**) 10Co, (**b**) 20Co, (**c**) 30Co, and (**d**) 35Co alloys, showing fcc or near-fcc single phase. Their average fcc grain sizes (D_fcc_) are listed below each map. All the alloys except for the 35Co alloy show a single fcc phase. The fraction of bcc phase in the 35Co alloy is about 0.5%.

**Figure 3:**
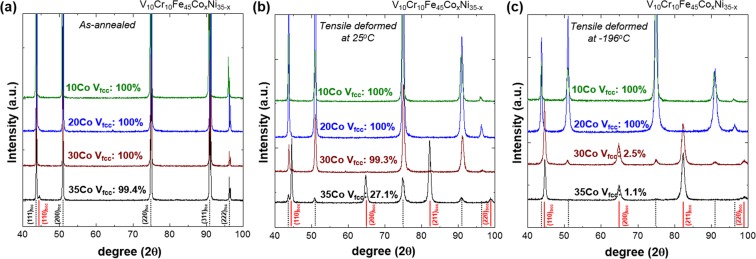
As-annealed and tensile-deformed microstructure. XRD patterns of the (**a**) as-annealed, (**b**) room-temperature tensile-deformed, and (**c**) cryogenic-temperature tensile-deformed HEAs. In the as-annealed state, all the alloys except for the 35Co alloy show a single fcc phase, and the 35Co alloy contains about 0.5% of bcc phase. After the room- and cryogenic-temperature tensile deformation, the V_fcc_ decreases with increasing Co content, which well satisfies the objective of the present MEA design, *i*.*e*., generation of deformation-induced martensitic transformation.

Phases in the as-annealed MEAs were identified by the EBSD and XRD analyses. Figure 2(a–d) shows EBSD phase maps of the 10Co, 20Co, 30Co, and 35Co alloys, and their average fcc grain sizes (D_fcc_) are listed below each map. All the alloys except for the 35Co alloy show a single fcc phase. The fraction of bcc phase in the 35Co alloy is about 0.5%, which corresponds to the phase diagram (Fig. 1(e)). XRD profiles of the as-annealed MEAs (Fig. 3(a)) show the same results with the EBSD data, confirming the first objective (to obtain a single fcc phase in the as-annealed state) of the present MEA design. The lattice parameters of the fcc phase calculated by using (111), (200), (220), (311), and (222) fcc peaks of the 10Co, 20Co, 30Co, and 35Co alloys are 0.3592, 0.3588, 0.3590, and 0.3592 nm, respectively.

Figure 3(b) show XRD profiles after the room-temperature tensile deformation. The fraction of fcc phase (V_fcc_) is 100% in the 10Co and 20Co alloys, which means no phase transformation. However, the 35Co alloy shows distinct peaks of bcc phase, and the residual V_fcc_ is 27.1%. After the cryogenic-temperature tensile deformation (Fig. 3(c)), the V_fcc_ is 100% in the 10Co and 20Co alloys, but decreases considerably with increasing Co content. Particularly in the 30Co and 35Co alloys, the V_fcc_ is only 2.5% and 1.1%, respectively. This change in V_fcc_ reveals that the fcc to bcc martensitic transformation occurred during the tensile deformation, which well confirms the second objective (to increase the thermal stability of bcc phase at low temperature, *i*.*e*., generation of deformation-induced martensitic transformation) of the present HEA design. In addition, the fraction of transformed bcc phase increases with increasing Co content after the tensile deformation at room and cryogenic temperatures (Fig. 3(c)). The stability of fcc phase thus decreases with increasing Co content. Considering that the deformation-induced martensitic transformation is affected by the Gibbs free energy difference between the parent and daughter phases^38^, this result corresponds to our MEA design approach based on ΔG_bcc-fcc_ (Fig. 1(e,f)).

### Room and cryogenic-temperature tensile behaviors

**Figure 4:**
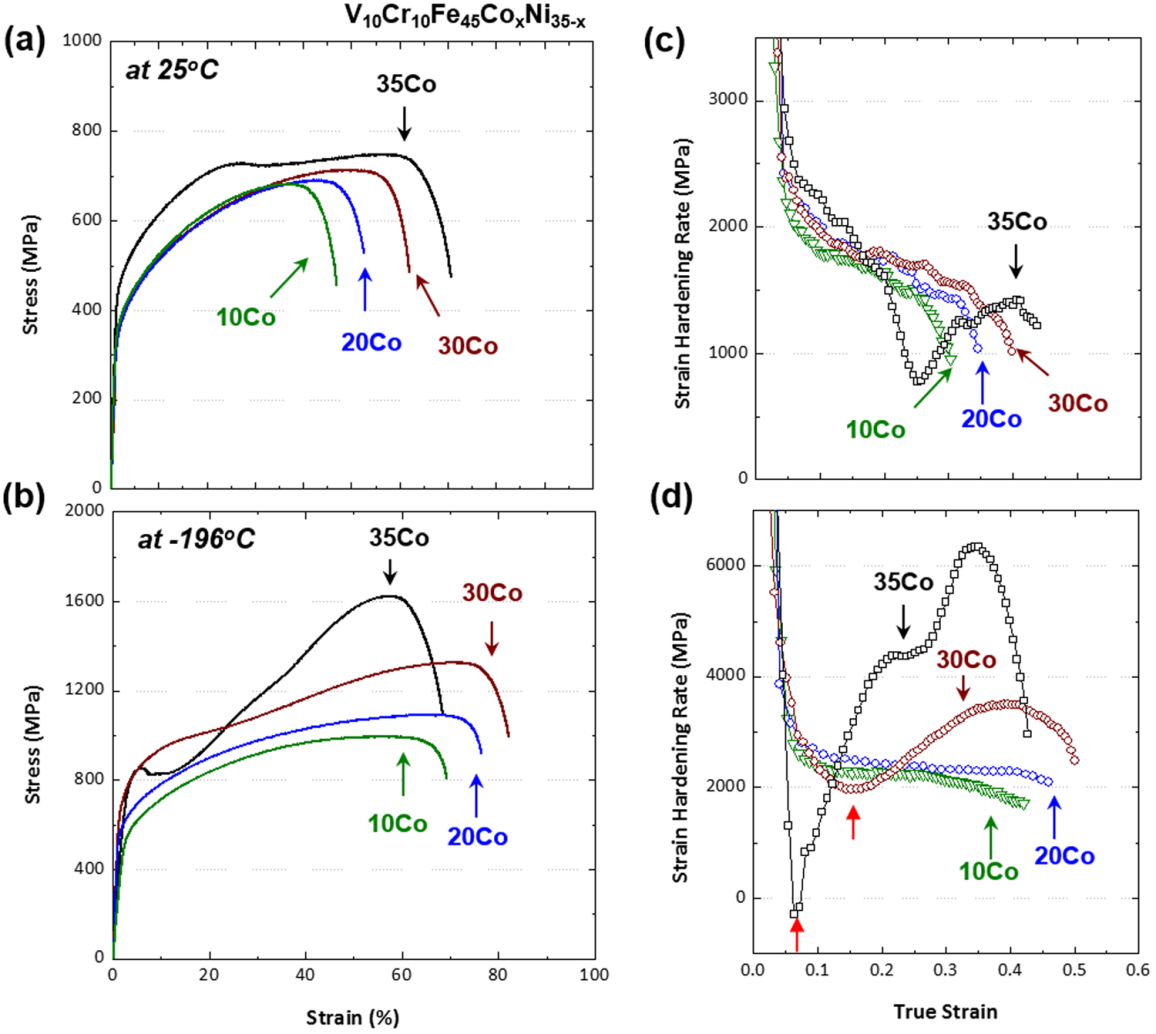
Tensile properties. Representative engineering stress-strain and strain hardening rate curves of the present MEAs tested at (**a**,**c**) room and (**b**,**d**) cryogenic temperatures. The MEAs exhibit the much higher strength and ductility as well as strain hardening capability at cryogenic temperature than at room temperature, although the ductility decreases by 5% in the 35Co alloy. Interestingly, the 35Co alloy shows the cryogenic-temperature tensile strength of 1623 MPa, which is twice higher than the room-temperature one. At cryogenic temperature, the 10Co and 20Co alloys show a gradually decreasing hardening rate, and the overall stain hardening rate tends to increase with increasing Co content, whereas the curves of the 30Co and 35Co alloys show the multiple-stage behavior.

Figure 4(a,b) shows representative engineering stress-strain curves tested at room and cryogenic temperatures, and the results are shown in Table 1. The MEAs exhibit the much higher strength and ductility as well as strain hardening capability at cryogenic temperature than at room temperature, although the ductility decreases by 5% in the 35Co alloy. Interestingly, the 35Co alloy shows the cryogenic-temperature tensile strength of 1623 MPa, which is twice higher than the room-temperature one. It is also noted that a change in stress-strain curves from a ‘parabolic’ shape to a ‘sigmoidal’ shape appears as the test temperature decreases^39^ or the Co content increases. The sigmoidal curve consists of an easy deformation stage (plateau in the stress-strain curve at lower strains) followed by a rapid hardening stage at higher strains^40,41^.

**Table 1:** Room (25 °C) - and cryogenic-temperature (−196 °C) tensile properties data of the V_10_Cr_10_Fe_45_Co_x_Ni_35−x_ MEAs.

Test Temp.	Alloy	Yield Strength (MPa)	Tensile Strength (MPa)	Elongation (%)
25 °C	10Co	339 ± 4.5	684 ± 4.2	47.0 ± 1.8
	20Co	345 ± 7.2	689 ± 5.2	52.6 ± 1.2
	30Co	348 ± 2.5	714 ± 3	62.0 ± 0.7
	35Co	427 ± 5.7	745 ± 4.9	70.1 ± 0.5
−196 °C	10Co	468 ± 3.5	996 ± 2.2	69.4 ± 0.5
	20Co	533 ± 8.5	1092 ± 3.2	76.3 ± 2.1
	30Co	601 ± 2.6	1291 ± 1.2	81.7 ± 4.2
	35Co	653 ± 5.6	1623 ± 1.0	65.0 ± 3.5

Figure 4(c,d) shows strain hardening rate curves tested at room and cryogenic temperatures. At room temperature, the 10Co, 20Co, and 30Co alloys show a decreasing trend of hardening rate, while their curves are not varied much (Fig. 4(c)). However, the curve of the 35Co alloy shows a multiple-stage behavior, *i*.*e*., down-up-down shape. At cryogenic temperature, the 10Co and 20Co alloys show a gradually decreasing hardening rate. In contrast, the curves of the 30Co and 35Co alloys show the multiple-stage behavior (Fig. 4(d)). The overall hardening rate tends to increase with increasing Co content, but the rates of the 30Co and 35Co alloys are lower than those of the 10Co and 20Co alloys in the initial deformation stage (red-arrow marks). This strain ‘softening’ is known to result from a deformation-induced austenite to ε-martensite transformation in metastable stainless steels^40,41^, and will be discussed later in the discussion part.

### Microstructural evolution during tensile deformation

**Figure 5:**
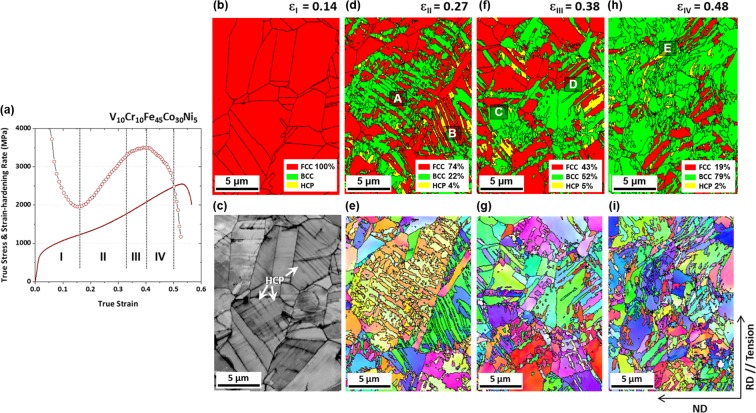
Cryogenic-temperature deformation mechanism of the 30Co alloy. (**a**) Cryogenic-temperature true stress-strain and strain hardening rate curves of the 30Co alloy. The strain hardening behavior is classified into Stages I through IV. (**b**–**i**) EBSD phase, IQ, and IPF maps demonstrating the fcc to bcc martensitic transformation in Stage I through IV in (**a**) (true strain range of ε_I_ to ε_IV_, *i*.*e*., 0.14~0.48). The phase transformation does not occur at a true strain of 0.14 (Stage I), although sharp parallel lines are observed inside fcc grains in IQ map. At a true strain of 0.27 (Stage II), 26 vol.% of fcc phase is transformed to bcc and hcp phases. Through Stages II and IV, the fcc to bcc transformation works as a dominant deformation mechanism.

The representative multiple-stage behavior of the 30Co alloy can be classified into Stages I through IV, as shown in Fig. 5(a). Outstanding cryogenic-temperature tensile properties are attributed to the continuously increased strain hardening in Stages II and III. The strain hardening rate is still higher than 2500 MPa in Stage IV, although the high strain rate reduces in further deformation. Here, we identify detailed deformation mechanisms and their contributions to mechanical response in each strain hardening stage, as shown in Fig. 5(b–i), for the 30Co alloy. All the EBSD phase and IPF maps were obtained from the central gage regions of specimens at each strain level.

Figure 5(b) shows an EBSD phase map at a true strain of 0.14 (Stage I). The phase transformation is not observed in the phase map of Stage I, although sharp parallel lines are observed inside fcc grains in image quality (IQ) map (Fig. 5(c)). In some grains, these lines are very populated and are crossed each other.

At a true strain of 0.27 (Stage II), 26 vol.% of fcc phase is transformed to the bcc and hcp phases (Fig. 5(d)). Some bcc phases are formed as complex network structures containing a plenty of high-angle grain boundaries or phase boundaries (marked by ‘A’ area) or along existing primary hcp phases (marked by ‘B’ area). The bcc phases have a similar crystallographic orientation (as shown in an inverse pole figure (IPF) map (Fig. 5(e)) because they are transformed from a parent fcc grain^42^. Yet, the bcc phases are not sufficiently coalesced yet in this Stage II. As the deformation stage proceeds from I to II, the fraction of bcc phase as well as high-angle grain boundaries or phase boundaries increases, thereby resulting in the rapid increase of strain hardening curve.

Figure 5(f–i) shows EBSD phase maps at true strains of 0.38 and 0.48 (Stages III and IV, respectively, in Fig. 5(a)). In Stage III, the V_fcc_ is 43% as 31 vol.% of fcc phase additionally transforms to bcc or hcp phase. The strain hardening rate also increases as the fraction of bcc phase increases, but the slope of hardening rate starts to decrease in Stage III. This is because the bcc phases are grown and coalesced (marked by ‘C’ and ‘D’ areas) and the effects of high-angle grain boundaries or phase boundaries are reduced. However, the TRIP occurs continuously so that the strain hardening rate reach 3600 MPa.

In Stage IV, some transformed bcc phases are deformed and bent as marked by ‘E’ area (Fig. 5(h,i)), resulting in the decrease of strain hardening rate (Fig. 5(a)). When the tensile deformation proceeds further to the tensile failure, only a 2.5 vol.% of fcc phase remains (Fig. 3(c)). The present high strain hardening rate and resultant outstanding tensile properties at cryogenic-temperature, therefore, are originated from the multiple deformation mechanisms: dislocation slip; transformation from the fcc to bcc phase; and a complex network structure containing a plenty of high-angle grain boundaries or phase boundaries.

**Figure 6:**
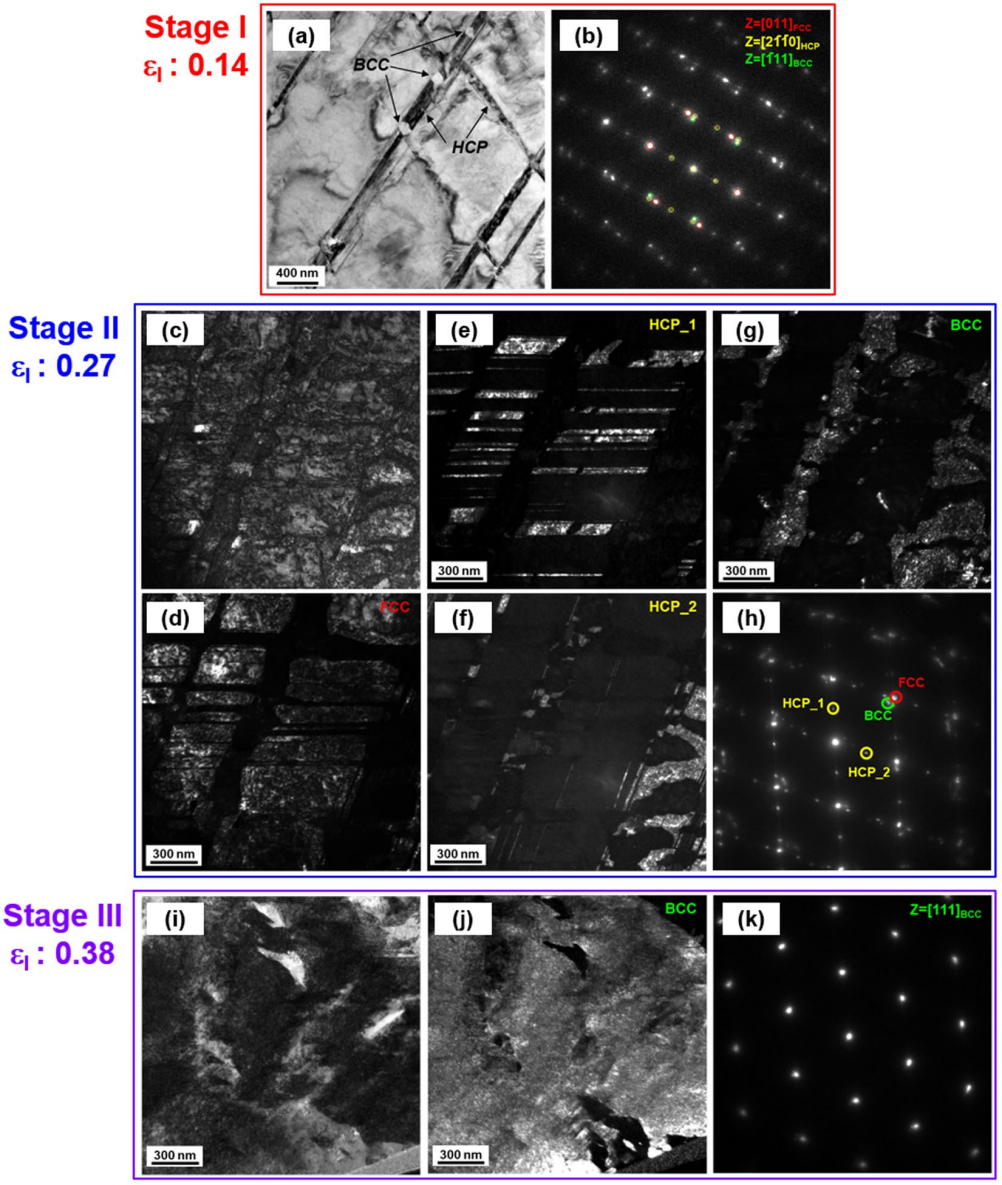
Cryogenic-temperature deformation mechanism of the 30Co alloy. (**a**,**b**) TEM bright-field image image and selected area diffraction (SAD) pattern at a true strain of 0.14 (Stage I). A bcc phase is nucleated at intersections of crossed primary and secondary hcp phases. (**c**–**h**) TEM bright-field image, dark-field image, and selected area diffraction (SAD) pattern at a true strain of 0.27 (Stage II). A bcc phase is grown along with hcp phases. (**i**–**k**) TEM bright-field image, dark-field image and selected area diffraction (SAD) pattern at a true strain of 0.38 (Stage III). A bcc phase coalesces.

TEM analyses were performed for the further detailed microstructural observation through Stage I to Stage III for the 30Co alloy. In Stage I, sharp parallel lines (arrow mark in Fig. 5(c)) are identified to be hcp phases by a selected area diffraction (SAD) pattern (Fig. 6(a,b)), which indicates the fcc to hcp martensitic transformation. It is also found that bcc phases are nucleated at intersections of primary and secondary hcp phases. Hcp and bcc phases have Shoji-Nishiyama ({111}_fcc_||{0002}_hcp_, <011>0_fcc_||<2-1-10>_hcp_) and Kurdjumov–Sachs ({111}_fcc_||{110}_bcc_, <011>_fcc_||<111>_bcc_) orientation relations, respectively, with fcc phase. Hcp phase has Burgers orientation relations of ({110}_bcc_||{0002}_hcp_, <111>_bcc_||<2-1-10>_hcp_) with bcc phase. Since the width and size of hcp and bcc phases are fine, they are not identified in the EBSD maps.

Figure 6(c–h) shows TEM results for the ‘A’-marked area in Fig. 5(d,e) by FIB sampling. In Stage II, the fcc phase is refined by the formation of hcp phases and their intersections (Fig. 6(d)). The primary and secondary hcp phases are observed (Fig. 6(e,f)), and the bcc phase is grown along with them (Fig. 6(g)). Figure 6(i–k) shows TEM results for the ‘D’-marked area in Fig. 5(f,g) by FIB sampling, which confirms the coalescence of bcc phases in Stage III.

## Discussion

The above results reveal that our design approach based on the difference in Gibbs free energies between bcc and fcc phases (ΔG_bcc-fcc_) can realize the fcc to bcc transformation in MEA research areas. The fraction of bcc and consequent transformation rate according to the Co content and temperature also well correspond to the thermodynamic phase stability. In this section, we discuss the microstructural evolution and phase stability, varied with the balance of Co and Ni, and their contributions to the mechanical response in detail.

### Microstructural evolution according to Co and Ni balance

**Figure 7:**
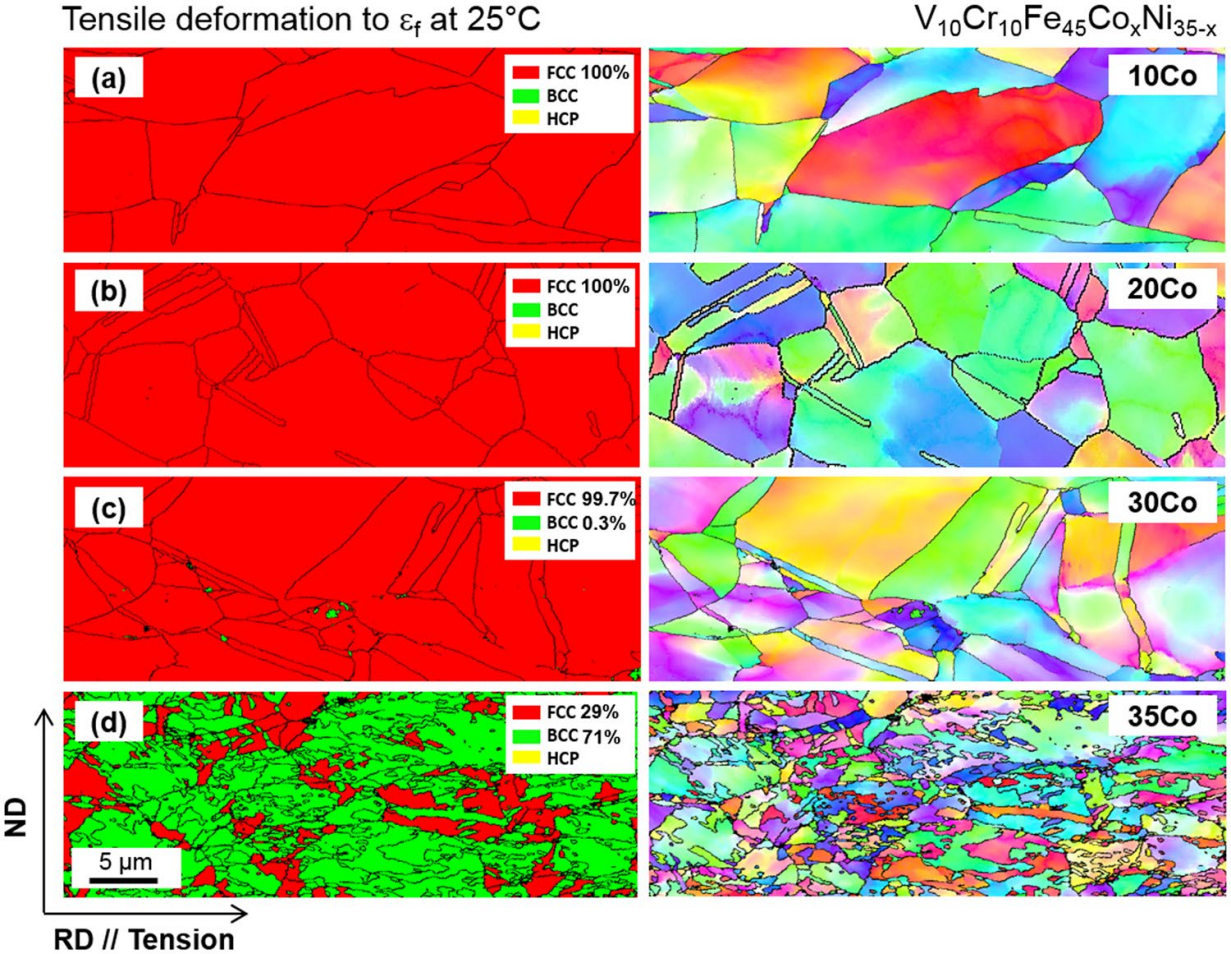
Room-temperature tensile deformation behavior. EBSD phase and IPF maps after the room-temperature tensile deformation of the (**a**) 10Co, (**b**) 20Co, (**c**) 30Co and (**d**) 35Co alloys. The 10Co and 20Co, and 30Co alloys show mainly the dislocation glide without the deformation twinning, and their martensitic transformation is negligible, whereas the 35Co alloy shows a high fraction of bcc transformation.

**Figure 8:**
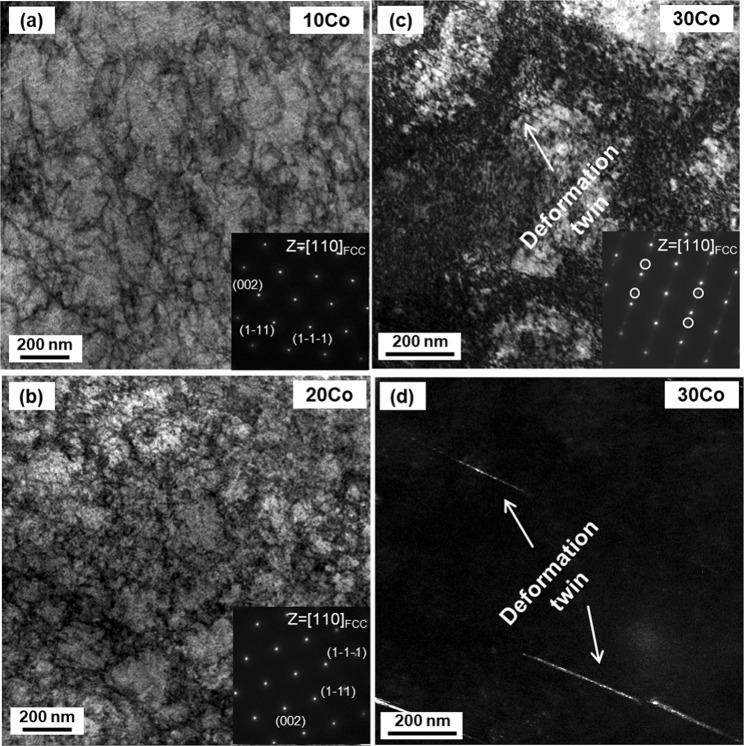
Room-temperature tensile deformation behavior. TEM bright-field image, dark-field image, and SAD patterns after the room-temperature tensile deformation of the (**a**) 10Co, (**b**) 20Co, and (**c**,**d**) 30Co alloys. The 10Co and 20Co alloys show tangled dislocations, while the 30Co alloy shows a small amount of deformation twins.

As confirmed by the XRD results (Fig. 3(b)), the phase transformation rarely occurs in all the alloys except for the 35Co alloy after the room-temperature tensile deformation. Additional EBSD analyses also confirm these results. Figure 7(a–d) shows EBSD phase and IPF maps of the uniformly elongated regions apart from the necking regions after the room-temperature tensile fracture of the four alloys. The 10Co, 20Co, and 30Co alloys mainly show the dislocation glide without the deformation twinning, and their martensitic transformation is negligible (Fig. 7(a–c)). In contrast, the 35Co alloy shows a high fraction of bcc transformation (Fig. 7(d)). In order to clarify the deformation mechanism, TEM analyses were conducted for the 10Co, 20Co, and 30Co alloys, and the results are shown in Fig. 8(a–d). The 10Co and 20Co alloys show tangled dislocations without deformation twins (Fig. 8(a,b)). The 30Co alloy, however, shows the existence of sharp deformation twins which are hard to be observed in the EBSD (Fig. 8(c,d)).

In conventional fcc alloys^6,43–45^, for example, the deformation twinning prevails at the SFE above 20 mJ·m^−2^, whereas the martensitic transformation mainly occurs at the SFE below 20 mJ·m^−2^. Thus, the variation in deformation behavior with the increase in Co content (Fig. 3(b,c)) is similar to that of conventional fcc alloys with the decrease in SFE^6^. Both strength and elongation increase in the order of the 10Co, 20Co, and 30Co alloys (Fig. 4(a)), while their strain hardening rates are similar (Fig. 4(c)). This can be explained by the extension of partial dislocations because the decrease in SFE generally induces the highly-sustained strain hardening and the resultant delay of necking^46^. However, the 35Co alloy shows a remarkable strain hardening in the initial deformation stage followed by a multiple-stage deformation behavior (Fig. 4(a,c)). This behavior results from the martensitic transformation, which also observed in the strain hardening rate curve at cryogenic temperature.

**Figure 9:**
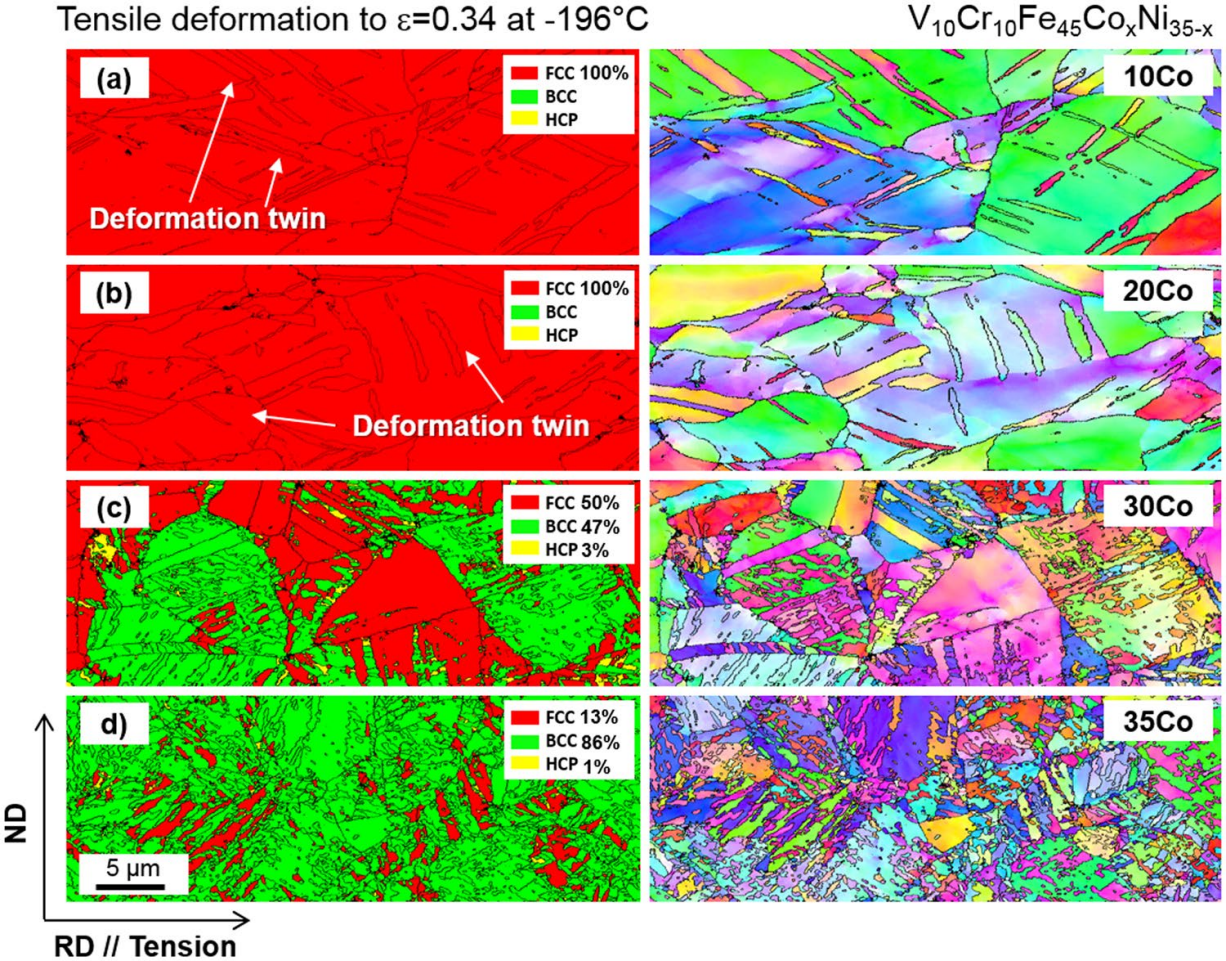
Cryogenic tensile deformation behavior. EBSD phase and IPF maps after the cryogenic-temperature tensile deformation to a true strain of 0.34 of the (**a**) 10Co, (**b**) 20Co, (**c**) 30Co, and (**d**) 35Co alloys. The 10Co alloy shows a transition of deformation mechanism from dislocation slip to deformation twinning and 20Co show a larger amount of deformation twinning as the temperature decreases from room temperature to cryogenic temperature. In the 30Co and 35Co alloys, most of fcc phases are transformed to bcc phases, but the 35Co alloy shows the higher fraction of bcc phase than the 30Co alloy.

**Figure 10:**
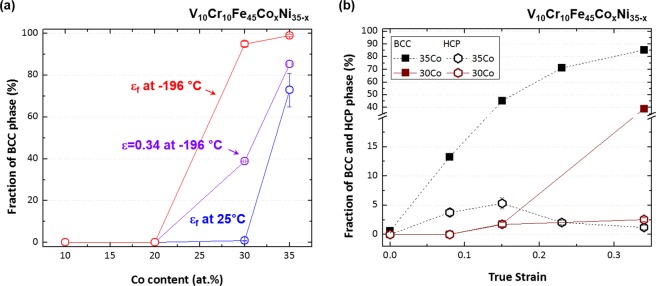
Volume fractions of bcc or hcp phase under room- and cryogenic-temperature tensile deformation. (**a**) Volume fraction of bcc phase as a function of Co content in the Fe_45_Cr_10_V_10_Co_x_Ni_(35−x)_ system tensile-deformed at room and cryogenic temperatures. Since the transformed bcc fractions correspond well to the thermodynamic approach, the Co-Ni balance plays a leading role in the phase stability optimization for the deformation-induced transformation. (**b**) Volume fractions of bcc and hcp phases as a function of true strain of the 30Co and 35Co alloys. The fcc phase in the 30Co alloy almost remains at the true strain of 0.08, but starts to transform to hcp and bcc phases at 0.15. In the 35Co alloy, a considerable amount of bcc and hcp phases (13.3% and 3.8%, respectively) is formed even at 0.08.

At cryogenic temperature, the microstructural evolution and phase stability according to the Co-Ni balance show similar trends to those at room temperature. The XRD results (Fig. 3(c)) indicate that the amount of bcc martensite increases as the Co content increases, but it is not varied much in the range of 97~99% for the 30Co and 35Co alloys. According to their stress-strain and strain hardening rate curves (Fig. 4(c,d)), however, the stability of fcc phase seems to be lower in the 35Co alloy than in the 30Co alloy. In order to carefully analyze the transformation rate in relation with the fcc stability, the EBSD analyses were conducted for the specimens tensile-deformed to a true strain of 0.34 at cryogenic temperature. EBSD phase and IPF maps were obtained from the central gage regions of specimens, as shown in Fig. 9(a–d). The 10Co and 20Co alloys show a transition of deformation mechanism from dislocation slip to deformation twinning as the temperature decreases from room temperature to cryogenic temperature (Figs 7(a,b) and 9(a,b)). Thus, the strain hardening rate decreases more slowly at cryogenic temperature than at room temperature (Fig. 4(c,d)). In the 30Co and 35Co alloys, most of fcc phases are transformed to bcc phases after the cryogenic-temperature tensile test (Fig. 3(c)), but the 35Co alloy shows the higher fraction of bcc phase (86%) than the 30Co alloy (47%) at the true strain of 0.34 (Fig. 9(c,d)). This indicates the higher transformation rate in the 35Co alloy than that in the 30Co alloy. Figure 10(a) summarizes the bcc fraction as a function of Co content, showing the stability of fcc decreases with increasing Co content and also with decreasing temperature. These bcc fraction curves demonstrate that the decrease of ΔG_bcc-fcc_ from −1300 to −5800 J·mol^−1^ with increasing Co content from 10 to 35 at.% (Fig. 1(f)) well confirms the reduced stability of fcc phase.

It is important to note that the martensitic transformation in this MEAs is also associated with the formation of hcp as well as bcc. Figure 10(b) exhibits fractions of bcc and hcp phases of the 30Co and 35Co alloys, measured by XRD analyses, as a function of tensile true strain. The fcc in the 30Co alloy almost remains at the true strain of 0.08, but starts to transform to hcp and bcc at 0.15. In the 35Co alloy, however, a considerable amount of bcc and hcp (13.3% and 3.8%, respectively) is formed even at 0.08. Note that a systematic error might be introduced in the XRD measurements. When the hcp fraction is very low, hcp reflections can be ignored in the quantification of volume fraction from XRD^1^.

**Figure 11:**
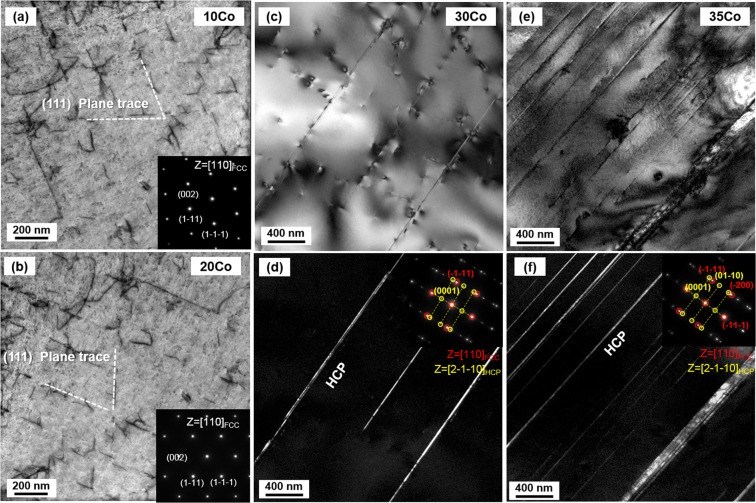
Initial cryogenic tensile deformation behavior. STEM images, Bright field images and selected area diffraction (SAD) patterns of the (**a**) 10Co, (**b**) 20Co, (**c**,**d**) 30Co, and (**e**,**f**) 35Co alloys tensile-deformed to a true strain of 0.04 at cryogenic temperature. The 10Co and 20Co alloys show a planar slip characteristic as dislocations are arrayed along (111) slip planes. In the 30Co and 35Co alloys, sharp parallel lines are observed, and are confirmed to be hcp phases by the SAD pattern analysis. The 35Co alloy shows a larger amount of hcp phase.

To avoid this argument, thus, the formation of hcp phase was further confirmed by the TEM analysis. Figure 11(a,b) shows bright-field scanning-TEM (STEM) images and SAD patterns of the 10Co and 20Co alloys tensile-deformed to a true strain of 0.04 at cryogenic temperature. Both alloys show a planar slip characteristic as dislocations are arrayed along (111) slip planes. Deformation twins or hcp formations are not observed. In the 30Co and 35Co alloys, sharp parallel lines are observed, and are confirmed to be hcp phases by the SAD pattern analysis (Fig. 11(c–f)). The 35Co alloy shows a more considerable amount of hcp phase, which is coincided with the XRD data (Fig. 10(b)).

In metastable high-Mn steels and austenitic stainless steels^6,21,39,41,47–49^, the martensitic transformation occurs through such various sequences as fcc→bcc, fcc→hcp→bcc, or fcc→hcp depending on the SFE and stability of fcc phase. The kind of transformation sequence of the 30Co and 35Co alloys belongs to the second one, however, the minor distinction with the alloys in previous literatures^6,22,48^ is the role of the hcp phase. The fraction of the hcp phase in the present MEAs is not high ( is~5%) as much as that of the bcc phase. In addition, the hcp phase formed at the initial deformation is almost consumed by the formation of bcc phase. Thus, the hcp phase is hard to play a significant role itself in overall strengthening mechanism, but acts mostly as a nucleation site for the bcc formation.

Regarding kinds of transformation mechanisms, it can be divided by stress- or strain-induced mechanism according to the fcc stability^47,50,51^. The stress-induced nucleation is activated under the sufficiently low stability by an elastic stress on pre-existing nucleation sites which are responsible for the athermal martensitic transformation during the cooling. That is, the yielding is dominated by the transformation. In contrast, the strain-induced transformation occurs at new nucleation sites created by intersections of microscopic shear bands, stacking faults, and deformation twins after the yielding. The hcp phase is formed at the initial deformation stage in both of the 30Co and 35Co alloys (Fig. 11(c–f)), which is associated with the stress-induced transformation. This hcp phase subsequently acts as a nucleation site for the bcc phase, indicating the fcc to bcc transformation is defined as a strain-induced transformation^52,53^.

### Contribution of phase stability to mechanical responses

The role of phase stability in TRIP mechanism is interesting, but the more important result is its contribution to the mechanical response. The strain hardening of the TRIP alloys, *e*.*g*., the 30Co and 35Co alloys, shows a multiple-stage behavior which is classified into Stages I through IV, as shown in Figs 4(d) and 5(a).

In Stage I, the decreased strain hardening rate which is usually resulting from dynamic recovery^54^ is mainly attributed to the fcc to hcp transformation. This strain ‘softening’ phenomenon has also been reported in metastable austenitic stainless steels^39^, Fe-Mn alloys^21,48^, and Fe-Mn-Si alloys^49^. Gunter and Reed^55^ proposed that the easy deformation (strain softening or plateau) and rapid hardening stage were closely related with the formation of ε-martensite and α’-martensite, respectively. The ε-martensite tended to appear at the easy deformation and disappear at the beginning of the rapid strain hardening increase stage^41,55^, which is related with the increased fraction of bcc martensite. Tamura *et al*.^56^ interpreted that strain softening was caused by the normal slip deformation and the deformation due to ε-martensite formation. Bhadeshia *et al*.^57^ reported that the strain softening was associated with the lattice volume differences between fcc and hcp phases. There can exist a contraction of 1~2% in atomic spacing along the direction perpendicular to (111)_fcc_ planes during the martensitic transformation. However, the fcc to hcp transformation occurs with a large transformation shear of 0.35. There is an almost no change in lattice volume between fcc and hcp phases in actual, therefore, leading to the strain softening or plateau in the stress-strain curve at low strains during the hcp formation.

The 35Co alloy shows the hcp transformation even at a true strain of 0.08 (3.8%, Fig. 10(b)). Thus, it is readily expected that the strain hardening rate at the initial deformation stage is gradually reduced by the formation of hcp phase until the formation of bcc phase is activated. Since the strain at which the hardening rate starts to increase is similar to that at which the bcc phase begins to form (Figs 4(d) and 10(b)), the suppression of the strain softening is attributed to the formation of bcc phase. The 35Co alloy which shows the higher fraction of hcp phase than that of the 30Co alloy, however, displays the severe extent of softening and the subsequent largely decreased strain hardening rate. This effect results in the stress drop after the yielding (Figs 4(b,d), 10(b) and 11(c–f)). This indicates that the fcc to bcc martensitic transformation is more effective for the strain hardening than the fcc to hcp transformation, as highlighted in the present research objectives.

In stage II, as the transformation from fcc to bcc occurs, a complex network structure containing a plenty of high-angle grain boundaries or phase boundaries creates additional obstacles against the dislocation slip, which reduces the mean free path of dislocations (Fig. 5(d,e)). The complex structure leads to the dynamic grain refinement and significant enhancement of strain hardening rate. Also, the fcc to bcc transformation is associated with a large dilatational shear strain (change in volume)^55–57^. This large transformation strain introduces a large number of mobile dislocations in the fcc matrix at the vicinity of martensitic grains^41^. Such large transformation strains with the formation of bcc phase thus leads to the extensive strain hardening rate. The increased strain hardening rate in Stage III can also be interpreted by the increased fraction of bcc phase. The difference between Stage III and Stage II is the decreased slope of hardening rate (Fig. 5(a)), which results from the growth and coalescence of bcc phase. It reduces the effects of high-angle grain boundaries or phase boundaries. In stage IV, the fcc phase is almost exhausted, and the strain hardening rate decreases.

The actual fractions of transformed bcc phase well correspond to the present thermodynamic approach. The transformation-evolved deformation mechanisms are effectively utilized as powerful mechanisms for the great improvement of tensile properties, particularly at cryogenic temperature, confirming the success of the present MEA design strategies. Among the strategies, our first design objectives, i.e., the maintenance of single fcc phase even at cryogenic temperature before the deformation is quite significant because the continuous transformation to near-single bcc phase can maximize the TRIP effect^38,44^. It is interesting to note that the bcc phase, which generally deteriorates the cryogenic ductility and induces a ductile to brittle transition5,6, can lead to an achievement of the excellent ductility in this TRIP MEAs. Our findings demonstrate that the fcc to bcc transformation can be an attractive route to a new HEA/MEA design strategy for improving cryogenic strength-ductility.

## Conclusions

1. A VCrFeCoNi MEA system was designed by utilizing thermodynamic calculations in consideration of phase stability to generate the fcc to bcc transformation. In the Fe_45_Cr_10_V_10_Ni_x_Mn_(35−x)_ system, 20 at.% or higher of Ni was required to obtain a single fcc phase, but the decrease in ΔG_bcc-fcc_ was not large. The increase in Co content dramatically reduced the ΔG_bcc-fcc_ in the Fe_45_Cr_10_V_10_Co_x_Mn_(35−x)_ system, but the fcc region was quite limited. The Fe_45_Cr_10_V_10_Co_x_Ni_(35−x)_ system, however, showed a considerable expansion of the fcc region. Also, the bcc stability could significantly increase with increasing Co content, which was well corresponded to our HEA design strategy.
2. After the room-temperature tensile deformation, the 10Co and 20Co alloys mainly showed the tangled dislocation, and the 30Co alloy showed a little of deformation twins. The martensitic transformation in three alloys was negligible, whereas the 35Co alloy showed a high fraction of bcc martensite. After the cryogenic-temperature tensile deformation, the 10Co alloy showed a transition of deformation mechanism from dislocation slip to deformation twinning. The increase of Co content led to the activation of TRIP and the consequent considerable decrease in fcc fraction, which was well corresponded to the decrease in thermodynamically calculated ΔG_bcc-fcc_.
3. The martensitic transformation at cryogenic temperature generated a multiple-stage mechanical response. The sigmoidal curve consisted of an easy deformation stage at low strain followed by a rapid strain hardening stage at higher strains. In the former stage, the strain hardening rate gradually decreased by the hcp formation. Then, the strain softening disappeared after the bcc phase was nucleated. This indicated that the fcc to bcc transformation was more effective for the strain hardening than the fcc to hcp transformation, as highlighted in the present research objectives.
4. Outstanding cryogenic-temperature tensile properties (*e*.*g*., yield strength; 653 MPa, tensile strength; 1623 MPa, elongation; 65% in the 35Co alloy) were attributed to the continuously increased strain hardening in the later deformation stages. Large transformation strain and complex network structure containing a plenty of high-angle grain boundaries or phase boundaries led to the dynamic grain refinement and significant enhancement of strain hardening rate. This high strain hardening rate and resultant strength-ductility balance could be tuned by the Co-Ni balance which played a leading role in phase stability for the deformation-induced transformation.

## Methods

### Fabrication

The HEAs were fabricated in a vacuum induction melting furnace after master alloys were prepared from commercially pure elements (purity of each raw material was 99.9% at least). Approximately 150 g of the master alloy was molten in a ZrO_2_-coated SiO_2_ crucible, and poured into a rectangular graphite module (inner size; 100 × 35 × 8 mm). Thereafter, it was cleaned in a 20% HCl Cl80% C_2_H_6_O solution. The ingot was encapsulated in evacuated quartz ampules and homogenized at 1100 °C for 6 hrs. The sand blasting was conducted to smoothen the rugged ingot surface, and the ingot was cold-rolled with a reduction ratio of 75% to produce a 1.5-mm-thick sheet. The alloy sheets were annealed at 900 °C for 10 min followed by water-quenching. The chemical compositions of the present HEAs measured by a wet-chemical analysis are shown in Table 2.

**Table 2:** Chemical compositions measured from wet-chemical analysis for the V_10_Cr_10_Fe_45_Co_x_Ni_35−x_ MEAs. (unit: atomic %).

Alloy	V	Cr	Fe	Co	Ni
10Co	9.82	9.72	45.42	10.04	25.01
20Co	9.80	10.21	45.20	20.37	14.43
30Co	9.78	9.53	45.48	30.14	5.08
35Co	9.83	9.58	45.45	35.14	0

### Microstructural characterization

Phases were identified by using X-ray diffraction (XRD, Cu Kα radiation, scan rate; 2° min^−1^, scan step size; 0.02°). Their volume fractions were quantified by a method proposed by Moser^58^ using integrated intensities of (110)α, (200)α, (211)α, and (220)α peaks for α′-martensite, (111)_γ_, (200)_γ_, (220)_γ_, (311)_γ_, and (222)_γ_ peaks for austenite, and (10–10)ε, (10–11)ε, and (10–12)ε peaks for ε-martensite. These α′-martensite, austenite, and ε-martensite are equivalent to bcc, fcc, and hcp phases, respectively, in the present MEAs. The separation of (222)_γ_ peak is caused by radiation doublet^59^. In order to quantify the fractions according to the tensile deformation, the whole gage regions were analyzed for the interrupted tensile specimens (Fig. 10(a,b)), and the remaining half regions after tensile tests were analyzed for the specimens deformed to the failure (Figs 3 and 10(a)). EBSD analysis (step size; 0.05 μm) was performed by using a field emission scanning electron microscope (FE-SEM, Quanta 3D FEG, FEI Company, USA), and the analysis data were interpreted by using an orientation imaging microscopy (OIM) analysis software. XRD and EBSD specimens were mechanically polished and then electro-polished in a 92% CH_3_COOH (92%) +O8% HClO_4_ solution at 32 V to avoid a local phase transformation during the mechanical polishing. The EBSD data was interpreted by an orientation imaging microscopy (OIM) analysis software provided by TexSEM Laboratories, Inc. Deformed microstructures were identified by using a transmission electron microscope (TEM, model; 2100, Jeol, Japan) at 200 kV. TEM thin foils were prepared by using focused ion beam (FIB, model; Quanta 3D FEG, FEI Company, USA). For the EBSD and TEM analyses, the central regions of the gage were observed in the case of the interrupted tensile specimens (Figs 5, 6, 8, 9 and 11). For the specimens deformed to the failure, the uniformly elongated regions apart from the necking regions were analyzed.

### Mechanical property tests

Rectangular dog-bone-shaped specimens (gage length; 6.4 mm, width; 2.5 mm, thickness; 1.5 mm, longitudinal orientation) were tensioned at room temperature (25 °C) and cryogenic temperature (−196 °C) at a crosshead speed of 6.4 × 10^−3^ mm·s^−1^ by a 100-kN-capacity universal testing machine (model; 8801, Instron, Canton, MA, USA). A low-temperature chamber (inner size; 50 × 40 × 38 cm) was used for the cryogenic-temperature test. The extensometer could not be used at liquid nitrogen temperature, so strains were determined indirectly using correction factor from the crosshead displacement. In this study, the initial length was measured by marking the gauge part of the specimen before the cryogenic tensile test. After the cryogenic tensile test up to the plastic instability, the length between the initial markings was measured using OM and the correction factor was obtained by comparing with the crosshead displacement data. The same method was applied to the tensile elongation at room temperature, which was in good agreement with the tensile elongation at room temperature obtained by digital image correlation (DIC).
